# Silver-Zinc Redox-Coupled Electroceutical Wound Dressing Disrupts Bacterial Biofilm

**DOI:** 10.1371/journal.pone.0119531

**Published:** 2015-03-24

**Authors:** Jaideep Banerjee, Piya Das Ghatak, Sashwati Roy, Savita Khanna, Craig Hemann, Binbin Deng, Amitava Das, Jay L. Zweier, Daniel Wozniak, Chandan K. Sen

**Affiliations:** Comprehensive Wound Center, Davis Heart & Lung Research Institute, The Ohio State University Wexner Medical Center, Columbus, Ohio, United States of America; University of North Dakota, UNITED STATES

## Abstract

*Pseudomonas aeruginosa* biofilm is commonly associated with chronic wound infection. A FDA approved wireless electroceutical dressing (WED), which in the presence of conductive wound exudate gets activated to generate electric field (0.3–0.9V), was investigated for its anti-biofilm properties. Growth of pathogenic *P*. *aeruginosa* strain PAO1 in LB media was markedly arrested in the presence of the WED. Scanning electron microscopy demonstrated that WED markedly disrupted biofilm integrity in a setting where silver dressing was ineffective. Biofilm thickness and number of live bacterial cells were decreased in the presence of WED. Quorum sensing genes *lasR* and *rhlR* and activity of electric field sensitive enzyme, glycerol-3-phosphate dehydrogenase was also repressed by WED. This work provides first electron paramagnetic resonance spectroscopy evidence demonstrating that WED serves as a spontaneous source of reactive oxygen species. Redox-sensitive multidrug efflux systems *mexAB* and *mexEF* were repressed by WED. Taken together, these observations provide first evidence supporting the anti-biofilm properties of WED.

## Introduction

‘Electroceuticals’ encompass therapeutic use of electric currents or fields with the objective to amend impaired biological functions [1]. Electrical therapy was used since the early 18^th^ century and has re-emerged into prominence after the 1980s with applications ranging from wound healing, pain management to cardiac arrest management and nervous disorders. The use of wireless micro-current stimulation represents an emerging component of electroceuticals that eliminate the drawbacks of the traditional systems namely, electrochemical instability, risk of infection and electrical burns, pain and irritation [2,3]. The use of electric current to inhibit bacterial growth was first reported in 1965 [4], followed by subsequent reports over the years [5–9]. Electro-stimulation may influence both Gram (−) and Gram (+) bacteria. Continuous μA-level currents show better antimicrobial activity compared to pulsed currents [10].

Bacteria thrive in nature in two major physiological states, as free-living or planktonic bacteria, or in matrix-embedded complex biofilm structures [11]. Biofilm formation, relies, in part, on a form of inter-bacterial communication known as quorum sensing (QS), in which small diffusible signaling molecules called autoinducers globally regulate gene expression. Using QS, bacterial populations may switch from the planktonic to biofilm form operating as a concerted, multi-cellular entity [12].

Biofilms play an important role in the ecology of the earth and the sustainability of human life. At the same time, the role of biofilms in the pathogenesis of some chronic human infections is also widely accepted [13]. Clinically, treatment of biofilms present a major challenge, because bacteria encased in such structures evade host immune responses [14–19] and are markedly more tolerant to antibiotics [14,20]. Importantly, pathogenic bacteria rapidly acquire resistance against novel pharmacological solutions [21–26]. Biofilm bacteria are encapsulated in an extracellular matrix, consisting of several components including polysaccharides, proteins and DNA [27] which act as a diffusion barrier between embedded bacteria and the environment thus retarding penetration of antibacterial agents [28]. Additionally, because of limited nutrient accessibility, biofilm-residing bacteria are in a physiological state of low metabolism and dormancy increasing their recalcitrance towards antibiotic agents [29].

Chronic wounds present an increasing socioeconomic problem. An estimated 1–2% of western population suffers from chronic ulcers [30,31]. Approximately 2–4% of the national healthcare budget in developed countries are spent on treatment and complications due to chronic wounds [32]. The incidence of non-healing wounds is expected to rise as a natural consequence of longer lifespan and progressive changes in public health pattern like rising obesity, diabetes, and cardiovascular disease. Non-healing skin ulcers are commonly complicated by biofilm infection. Among the wound biofilm microbiome, *Pseudomonas aeruginosa* is known to play a major role delaying healing of leg ulcers [33]. Furthermore, surgical success with split graft skin transplantation and overall healing rate of chronic venous ulcers is lowered in the presence of *P*. *aeruginosa* [34].

In our previous work we have characterized the chemical composition and electric field generated by a wireless silver/zinc bioelectric wound dressing (Ag/Zn WED) [35]. In addition, we have reported on the mechanisms by which WED may accelerate keratinocyte migration and facilitate wound closure [35]. WED is FDA approved and is currently used as a wound dressing on our patients at the Comprehensive Wound Center. The objective of this work was to test the anti-biofilm properties of the wound dressing. WED is easy to handle and can be cut to the shape of the area of application and the electric field generated is proven to be safe for patients.

## Material & Methods

### Ag/Zn Bioelectric dressing

A bioelectric dressing, Procellera designed and provided by Vomaris Innovations, Inc. was used. A polyester cloth printed with polyvinylchloride with the same design was used as a control [35] The Ag control was the same polyester cloth with only Ag printed on it.

### Bacterial growth curves

*P*.*aeruginosa* (PAO1) was cultured in round bottom tubes in LB medium with continuous shaking @300rpm at 37°C. Absorbance was obtained using a spectrophotometer by measuring optical density at 600nm over different time points.

### In-vitro biofilm model

PAO1 biofilm was developed in vitro using a polycarbonate filter model. Grown overnight in LB medium at 37°C bacteria were cultured on sterile polycarbonate membrane filters placed on LB agar plates and allowed to form a mature biofilm for 48h. The biofilm was then exposed to WED or placebo for the following 24h [36].

### Energy Dispersive X-ray Spectroscopy (EDS)

EDS elemental analysis of the Ag/ZN WED was performed in an environmental scanning electron microscope (ESEM, FEI XL-30) at 25kV. A thin layer of carbon was evaporated onto the surface of the dressing to increase the conductivity.

### Scanning electron microscopy

Biofilm was grown on circular membranes and was then fixed in a 4% formaldehyde / 2% glutaraldehyde solution for 48 hours at 4°C, washed with phosphate-buffered saline solution buffer, dehydrated in a graded ethanol series, critical point dried, and mounted on an aluminum stub. The samples were then sputter coated with platinum (Pt) and imaged with the SEM operating at 5 kV in the secondary electron mode (XL 30S; FEG, FEI Co., Hillsboro, OR) [37].

### Live/Dead staining

The LIVE/DEAD *Bac*Light Bacterial Viability Kit for microscopy and quantitative assays was used to monitor the viability of bacterial populations. Cells with a compromised membrane that are considered to be dead or dying will stain red, whereas cells with an intact membrane will stain green.

### EPR spectroscopy

EPR measurements were performed at room temperature using a Bruker ER 300 EPR spectrometer operating at X-band with a TM 110 cavity. The microwave frequency was measured with an EIP Model 575 source-locking microwave counter (EIP Microwave, Inc., San Jose, CA). The instrument settings used in the spin trapping experiments were as follows: modulation amplitude, 0.32 G; time constant, 0.16 s; scan time, 60 s; modulation frequency, 100 kHz; microwave power, 20 mW; microwave frequency, 9.76

GHz. The samples were placed in a quartz EPR flat cell, and spectra were recorded at ambient temperature (25°C). Serial 1-min EPR acquisitions were performed. The components of the spectra were identified, simulated, and quantitated as reported [38,39]. The double integrals of DIPPMPO experimental spectra were compared with those of a 1 mM TEMPO sample measured under identical settings to estimate the concentration of superoxide adduct [40].

### Quantification of mRNA and miRNA Expression

Total RNA, including the miRNA fraction, was isolated using Norgen RNA isolation kit, according to the manufacturer's protocol. Gene expression levels were quantified with real-time PCR system and SYBR Green (Applied Biosystems) and normalized to nadB and proC as housekeeping genes. Expression levels were quantified employing the 2 (−ΔΔct) relative quantification method [41].

### Quantification of biofilm formation

Briefly, 2 ml of LB with low salt in 16 ml polystyrene tubes was inoculated with 10 μl of overnight bacterial suspension. The tubes were left undisturbed at 28°C and observed for biofilm-development and adherence to the tubes' wall. Thereafter, the liquid phase was discarded by inverting the tubes carefully. The tubes were air dried and heat fixed at 60°C for 1 hour. Subsequently, 300 μl of 100% methanol was added to each tube and left at room temperature for 15 min, with intermittent rotation at 5 min intervals to cover all the contents of adhered biofilm-mass with methanol. The methanol was replaced with 300 μl of crystal violet (1% solution in 50% methanol) and left for 10 minutes at room temperature with the rotations as described earlier. Finally, the stained contents were rinsed thoroughly with tap water. The tubes were air dried. For quantification of the adherent biofilm, the bound crystal violet was dissolved in 500 μl of 30% acetic acid and OD_570_ were noted [42–44].

### Aggregation assay

Aggregation was examined by diluting overnight cultures with fresh LB medium in 2.5 mL screw-capped tubes from 0% (no added fresh LB medium) to 100% (pure fresh LB medium). Cells were inverted gently several times and placed at room temperature for 15 min.

### Measurement of Pyocyanin in the supernatant of *P*. *aeruginosa*

Pyocyanin production by *P*. *aeruginosa* strains in the LB medium after 3 days was measured by taking 200 μl of bacterial cell free supernatant in 96-well microtiter plates and absorbance was recorded at 691 nm (λ_max_ of pyocyanin) using a microplate reader [45,46].

### Glycerol-3-phosphate dehydrogenase assay

Glycerol-3-phosphate dehydrogenase assay was performed using an assay kit from Biovision, Inc. following manufacturer’s instructions. Briefly, cells (∼1 x 10^6^) were homogenized with 200 μl ice cold GPDH Assay buffer for 10 minutes on ice and the supernatant (equal amount of protein loaded for control and treated) was used to measure O.D. and GPDH activity calculated from the results.

### Statistics

Control and treated samples were compared by paired *t* test. Student's *t* test was used for all other comparison of difference between means. *p* < 0.05 was considered significant. Data represented in bar graphs is plotted as mean ±SD. All replicates are biological replicates.

## Results

### Antimicrobial properties of Ag/Zn WED

**Fig 1:**
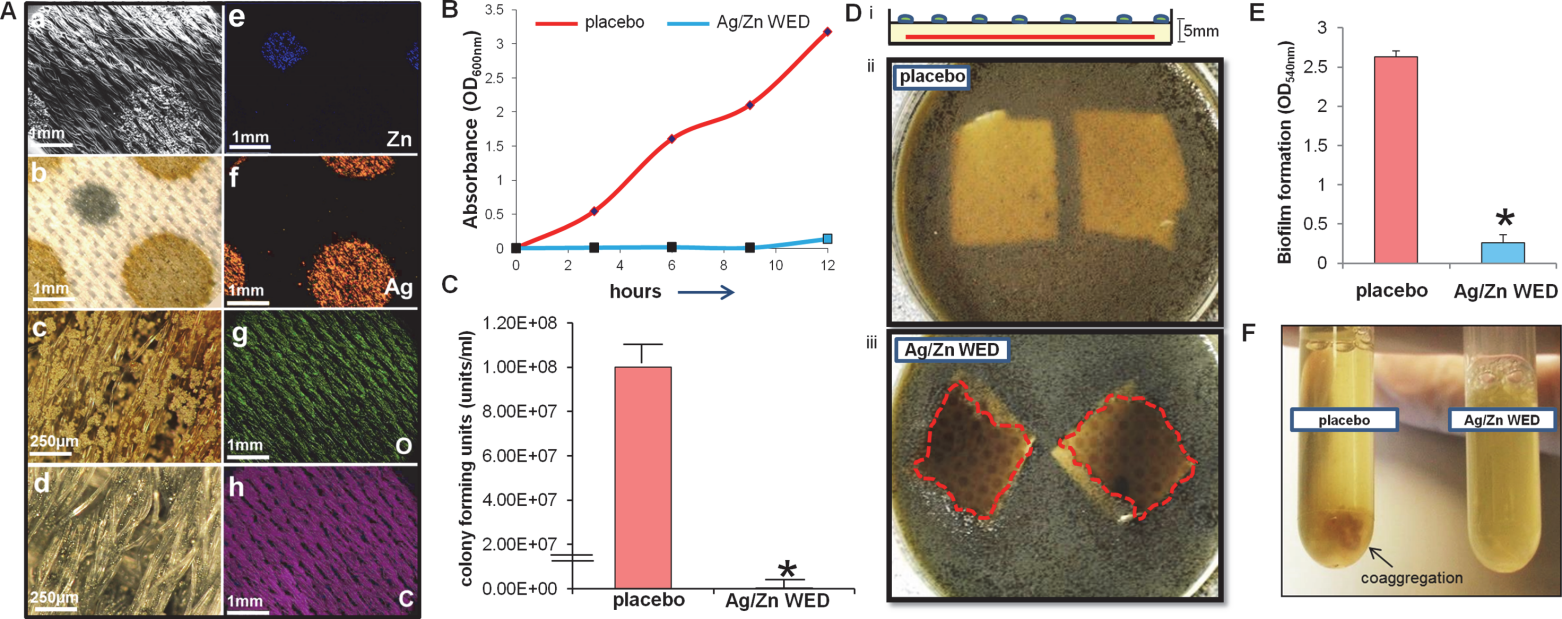
Anti-bacterial properties of WED. **(A)** Energy Dispersive X-Ray Spectroscopy (EDS) elemental analysis of Ag/Zn WED. **a.** Scanning Electron Microscope (SEM) image; **b.** Light Microscope Image; **c**. and **d.** Close-up view of gold and silver dots under light microscope. **e-h.** EDS element maps of Zn (blue), Silver (red), Oxygen (green) and Carbon (Magenta). **(B,C)** Absorbance and CFU measurement from planktonic PAO1 culture in presence of placebo or Ag/Zn WED, n = 4 **(D) (i)** schematic diagram for experimental design showing dressing embedded in the agar plate. **(ii, iii)** Zone of inhibition above WED is marked with red dotted line, while no such zone was observed over the placebo dressing, n = 4 **(E)** Biofilm formation measured by absorbance at 540 nm, n = 4 **(F)** Biofilm co-aggregation observed in the placebo treated overnight PAO1 culture was not observed in WED treated overnight cultures, n = 3.

High resolution electron micrographs were collected using an environmental scanning electron microscope (ESEM). Unlike conventional SEMs which require high vacuum in the specimen chamber, the ESEM can be run in a “high-pressure” environmental mode allowing the examination of hydrated or insulating samples [47]. The element maps generated by ESEM indicated that silver particles are concentrated in the dots with golden appearance. Zinc particles were noted to be concentrated in the dots with grey appearance **(Fig. 1A).** WED severely inhibited bacterial growth, (n = 4, *p*<0.001) **(Fig. 1B, C)**. When PAO1 was grown on an agar plate with control or Ag/Zn WED dressing embedded in the agar, the zone of clearance was clearly visible in the region under which WED was embedded thus demonstrating its bacteriostatic property (n = 4) **(Fig. 1D)**. This inhibition was observed up to a distance of 5 mm between the dressing and the top of the agar and disappeared at greater distances, indicating that the inhibition was electric field dependent which weakens over distance.

### Ag/Zn WED disrupted bacterial biofilm

**Fig 2:**
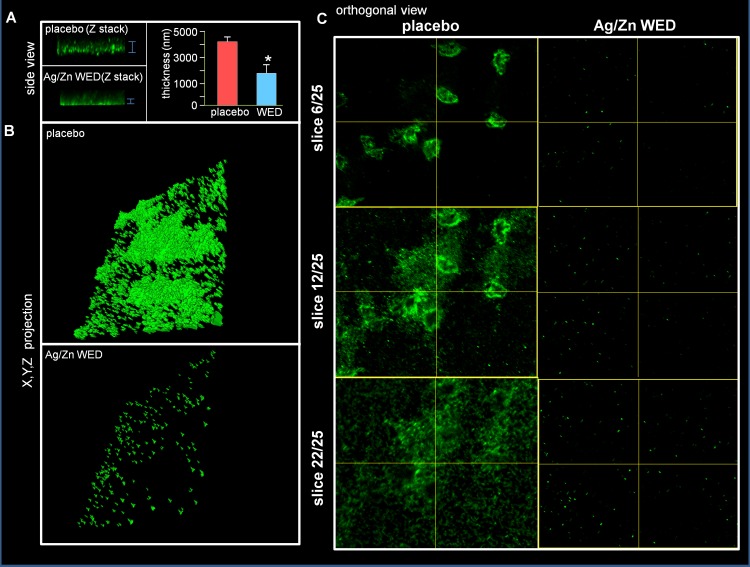
PAO1 staining demonstrates impaired biofilm formation by WED. **(A)** Side-view and thickness of in-vitro static mature biofilm treated with placebo or WED, n = 3 **(B)** X,Y,Z projection of same **(C)** Orthogonal view of same at slices 6, 12 and 22 out of total 25 slices acquired.

**Fig 3:**
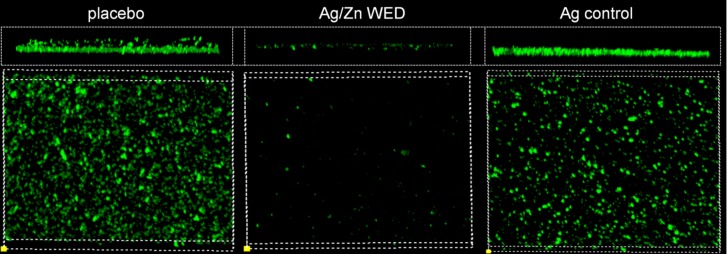
WED impairs extracellular polymeric substance (EPS) production. CLSM images of in-vitro static mature PAO1 biofilm grown treated with placebo, WED or Ag control and stained with EPS antibody, n = 3.

**Fig 4:**
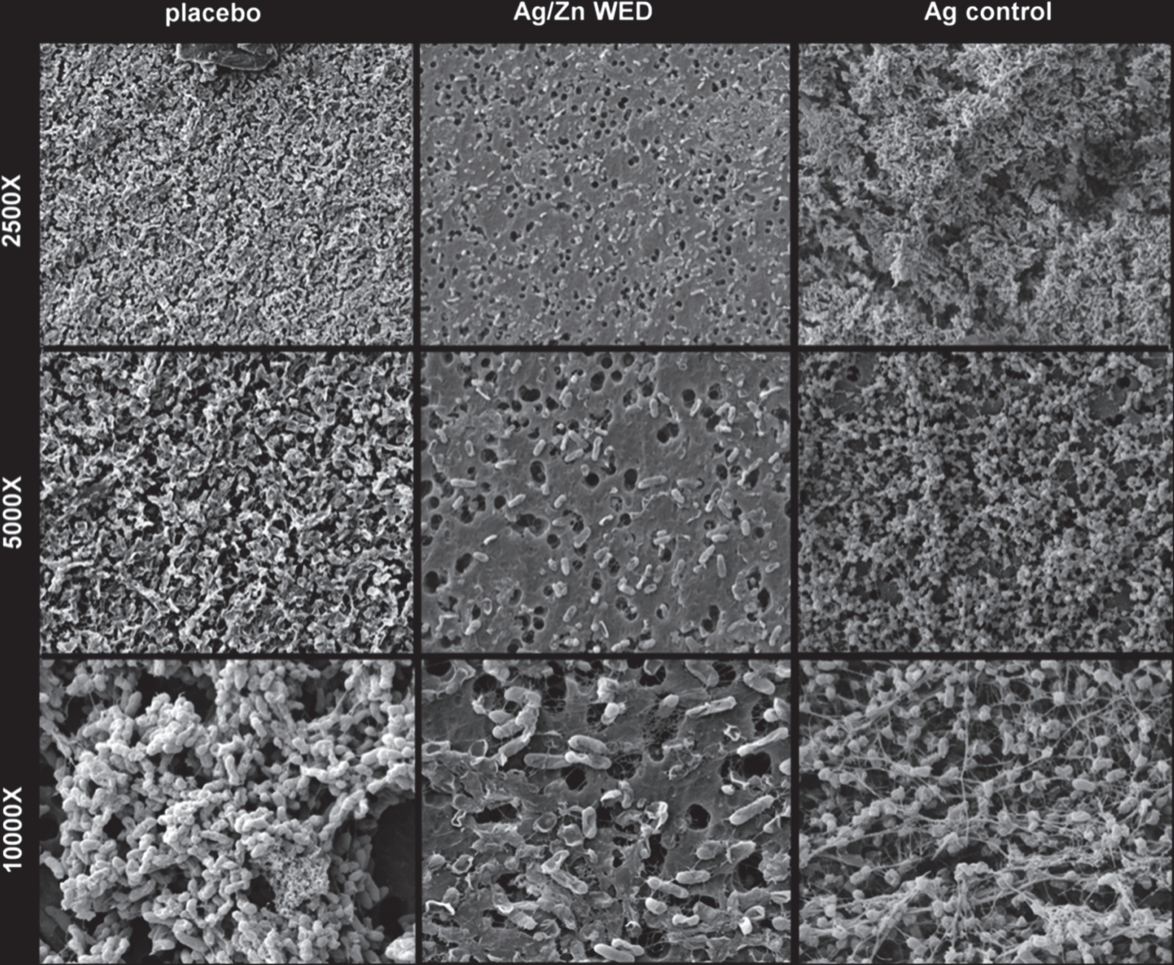
WED impairs biofilm structural integrity. Scanning Electron Microscope images of in-vitro static mature PAO1 biofilm treated with placebo, WED or Ag control shown at 2500X, 5000X and 10000X magnifications, n = 3.

**Fig 5:**
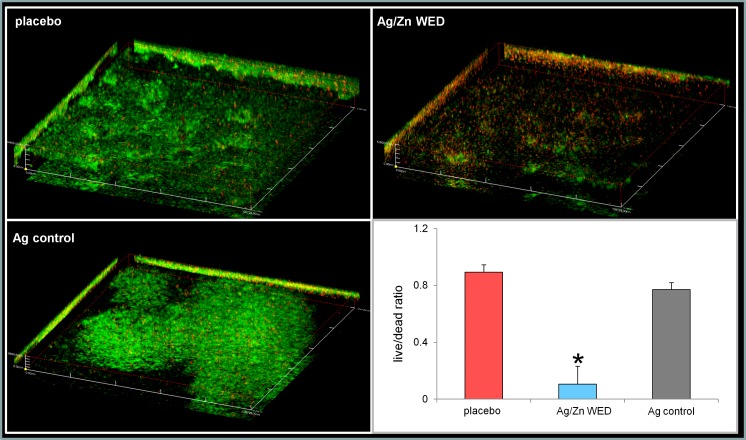
WED impairs cell viability. CLSM micrographs of mature PAO1 biofilm stained with live-dead stain after treatment with placebo, WED or Ag control. The green fluorescence indicates live bacteria while the red indicates dead bacteria, n = 3.

Attachment, motility and co-aggregation are some of the characteristics of bacterial biofilms. Ag/Zn WED inhibited biofilm formation on polystyrene surfaces (n = 4, *p*<0.05) **(Fig. 1E)**. Bacteria from biofilm communities exhibit co-aggregation, a specific mechanism of bacterial cell-to-cell adhesion that plays a key role in biofilm formation [48–50]. We observed that the WED significantly attenuated cell aggregation (n = 4) **(Fig. 1F)**. To further investigate the biofilm disruption properties of WED, scanning electron microscopy (SEM) and confocal laser scanning microscopy (CLSM) were utilized to visualize biofilm formation. PAO1 staining clearly demonstrated absence of biofilm like structures and decrease in thickness of the biofilm in the presence of the WED, (n = 3, *p*<0.05) **(Fig. 2).** Extracellular polymeric substance (EPS) formation was significantly impaired in the presence of WED (n = 3) **(Fig. 3)**. To further characterize the structural integrity of the biofilm, we employed SEM to visualize the biofilm. Marked disruption of bacterial biofilm structure was observed in the presence of the WED (n = 3) **(Fig. 4).** WED also caused bacterial cell death as observed by a live/dead staining of the biofilm grown in the presence of WED or placebo dressing (n = 3, *p*<0.05) **(Fig. 5)**. When the outcomes were compared to biofilm exposed to a silver-only control dressing, no significant effect on biofilm integrity, EPS or cell viability was observed **(Figs. 3–5)**.

### Ag/Zn WED generates superoxide and represses redox-sensitive multidrug efflux system in *P*. *aeruginosa*

**Fig 6:**
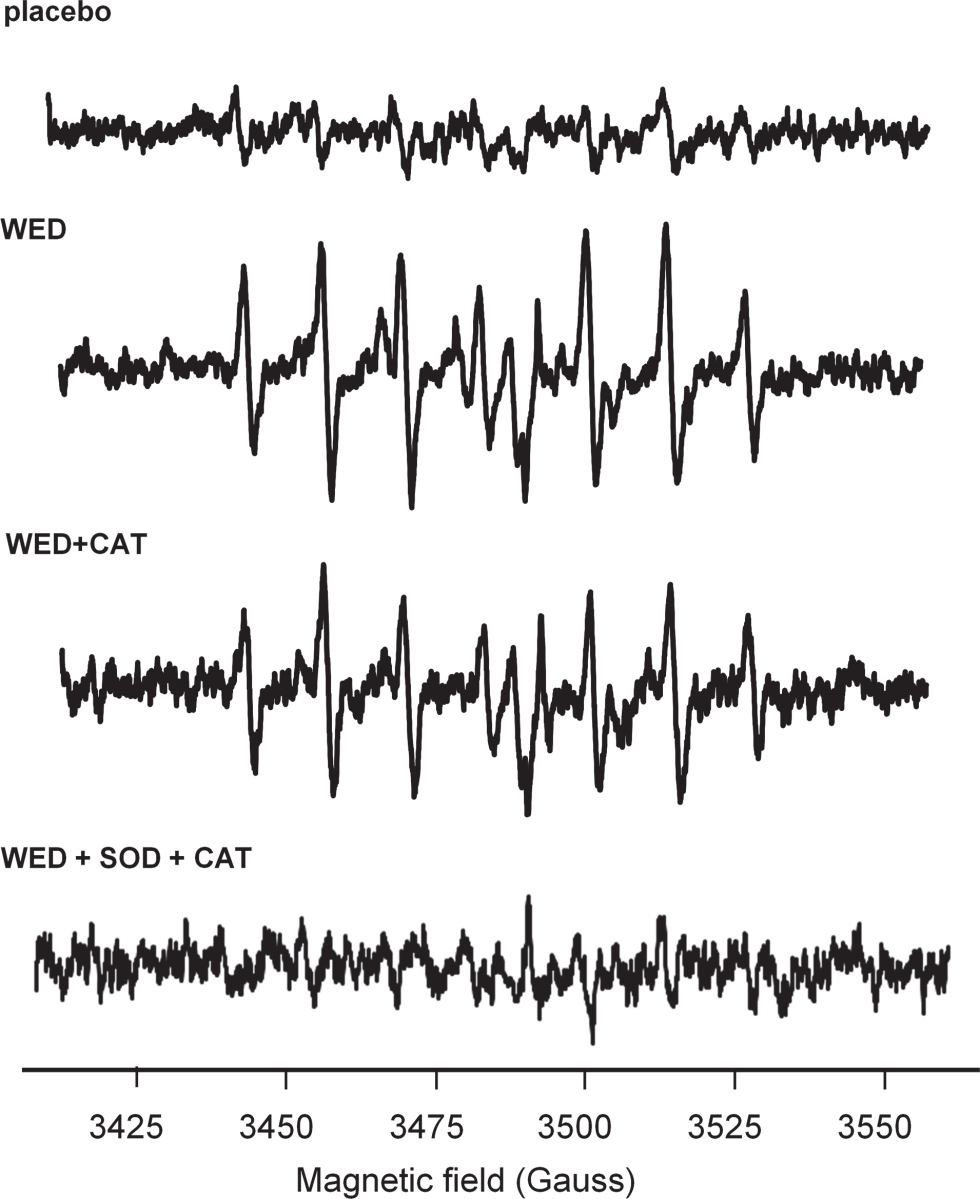
WED generates superoxide (A) EPR spectra using 20mM DIPPMPO demonstrates spin adduct generation upon exposure to dressings for 40 mins in PBS. Addition of 500U Catalase does not attenuate EPR signal but is attenuated using both 500U SOD and 500U Catalase, n = 3

In our previous work, we have demonstrated that Ag/Zn WED may serve as electron donor or reductant [35]. In this work we provide first direct evidence demonstrating that WED may result in one-electron reduction of oxygen to superoxide anion radical. Electron paramagnetic resonance (EPR) detected spontaneous superoxide production (∼1μM) by WED in phosphate buffered saline at room temperature, (n = 3, *p*<0.05) (**Fig. 6)**. Superoxides are known to rapidly dismutate to hydrogen peroxide. The EPR peaks were not attenuated in the presence of catalase or superoxide dismutase alone, but were blunted when both were used together.

**Fig 7:**
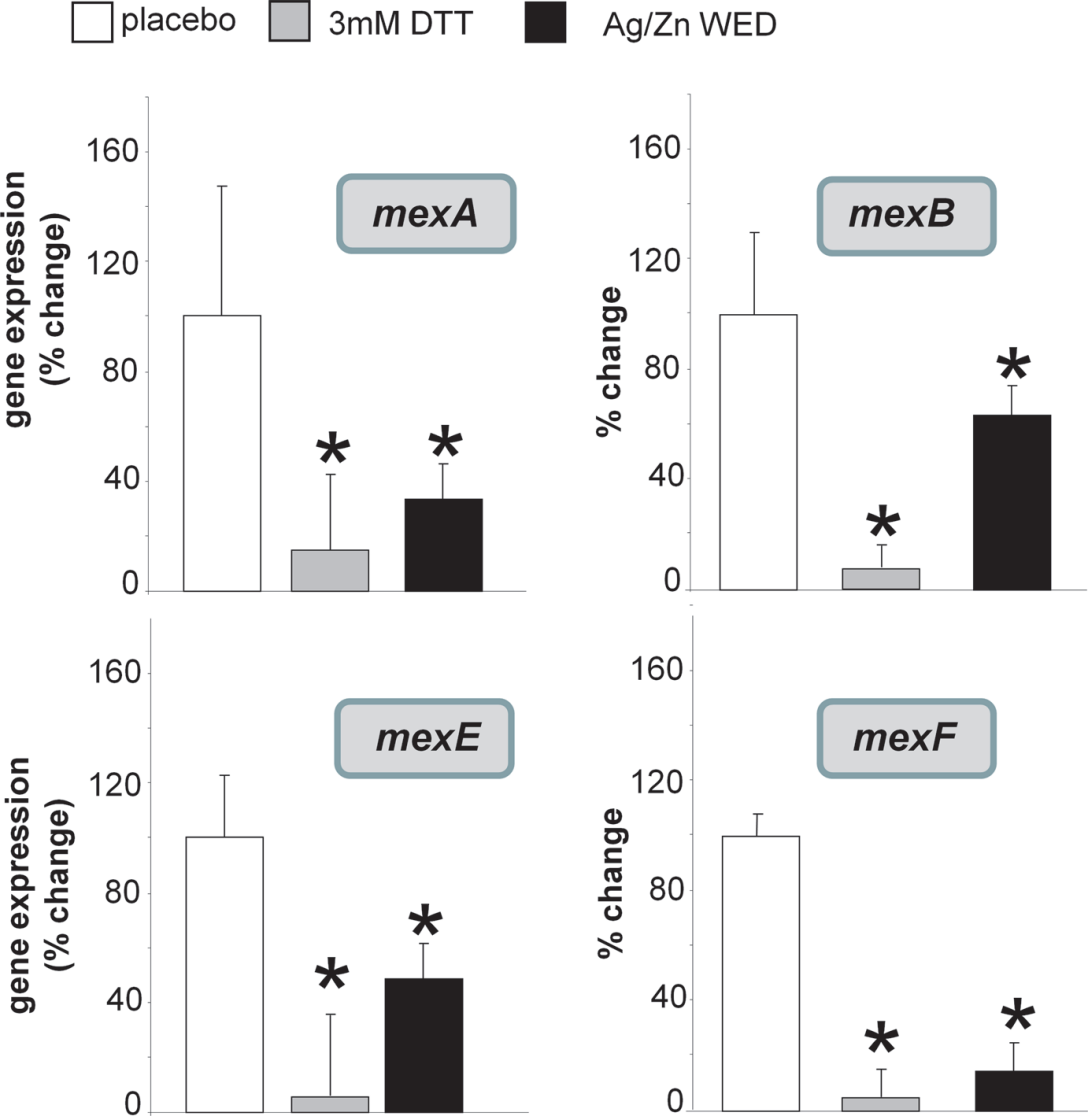
WED represses redox-sensitive multidrug efflux genes in *P*. *aeruginosa* Real-time PCR was performed to assess mex gene expression post-treatment with placebo, WED or 3mM DTT, n = 3.

MexR and MexT belong to the efflux pump virulence system in *P*. *aeruginosa* and are under the redox control of intermolecular disulfide bonds [24,51,52]. WED downregulated *mexA*, *mexB*, *mexE* and *mexF*, (n = 3, *p*<0.05). These genes are downstream of MexR and MexT and are expressed under conditions of oxidation. Reduced environment as caused by WED **(Fig. 6)** or DTT consistently blocked MexR/MexT dependent gene expression **(Fig. 7)**.

### Quorum sensing genes and glycerol-3-phosphate dehydrogenase activity in response to Ag/Zn WED

**Fig 8:**
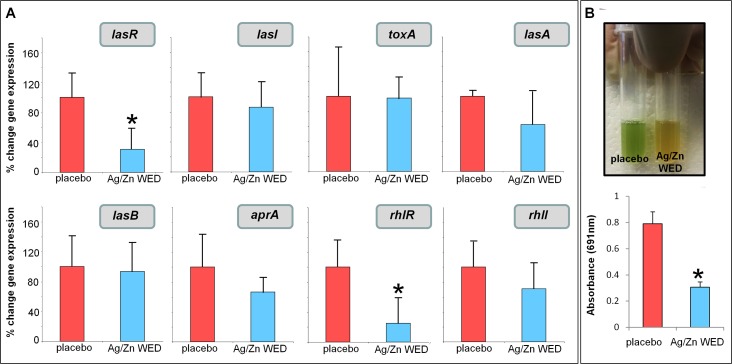
WED silences quorum sensing genes and pyocyanin production. **(A)** Real-time PCR was performed to assess quorum sensing gene expression with RNA isolated from mature PAO1 biofilm exposed to placebo or WED. *rpoD* and *proC* was used as housekeeping control, n = 3. **(B)** Pyocyanin production (green color) in overnight PAO1 culture followed by exposure to placebo or WED, n = 3.

**Fig 9:**
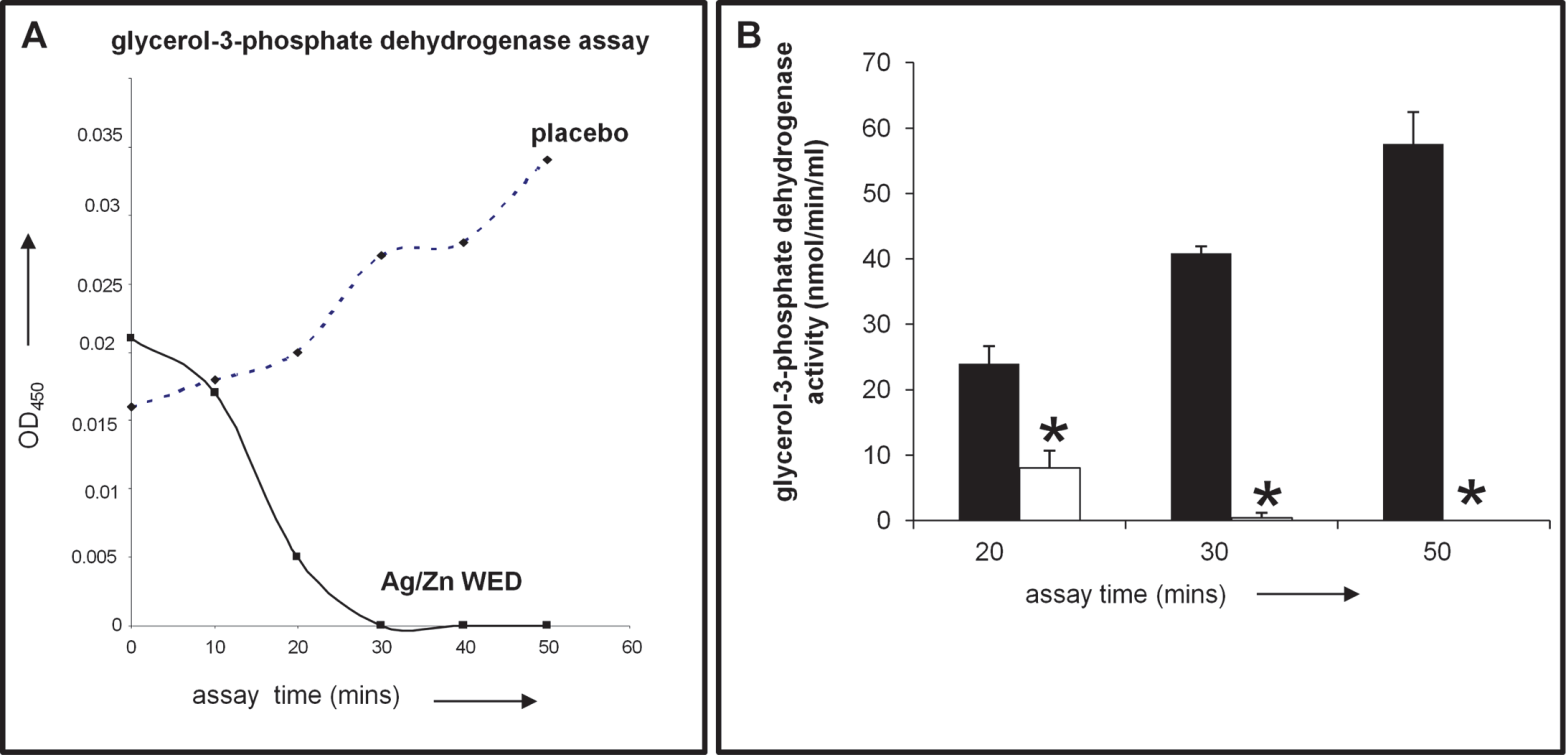
WED inhibits glycerol-3-phosphate dehydrogenase activity. Measurement of glycerol-3-Phosphate dehydrogenase enzyme activity from mature PAO1 biofilms treated with placebo or WED **A.** OD measured in the kinetic mode. **B.** GPDH activity calculated using the formula: Glycerol-3-Phosphate dehydrogenase activity = B/(ΔT X V) x Dilution Factor = nmol/min/ml, where: B = NADH amount from Standard Curve (nmol). ΔT = reaction time (min). V = sample volume added into the reaction well (ml), n = 3.

Expression of specific genes is known to be sensitive to electric field [53–57]. Exposure of WED on *P*. *aeruginosa* mature biofilm for 12h lowered levels of *lasR and rhlR*, (n = 3, *p*<0.05) **(Fig. 8A).** LasR and RhlR support the biosynthesis of pyocyanin by binding directly to the phenazine biosynthetic operons [58]. WED decreased pyocyanin production in PAO1, (n = 3, *p*<0.05), an observation that is consistent with lower *lasR and rhlR* **(Fig. 8B)**. Electrosensitive glycerol-3-phosphate dehydrogenase (GPDH) supports biofilm formation by *P*. *aeruginosa* [59,60]. WED attenuated GPDH activity of *P*. *aeruginosa*, (n = 3, *p*<0.05) **(Fig. 9).** In order to compensate for the reduced number of bacteria in the Ag/Zn WED treated samples due to killing and reduced growth, all gene expression studies were normalized to *proC* and *rpoD* as internal controls, which are known to be highly stable [61] and GPDH assay was normalized by loading equal amounts of protein.

## Discussion

Endogenous DC electric fields are important, fundamental components of development, regeneration, and wound healing [62]. Electroceuticals have sparked renewed interest in the health care industry [63]. This work presents the first evidence demonstrating that pathogenic bacterial biofilm may be disrupted using an electroceutical approach. The wireless electroceutical dressing (WED), used in this work, consists of a silver and zinc redox couple which may be printed on any textile surface [35]. Silver, by itself, is known to have antimicrobial properties [64]. However, the bactericidal concentration of silver required to eradicate the bacterial biofilm in chronic wounds has been found to be 10–100 times higher than that used to eradicate planktonic bacteria [65]. Thus, at non-cytotoxic levels silver has limited efficacy against bacterial biofilm [65,66]. Our observation that silver alone was unable to disrupt *P*. *aeruginosa* biofilm is consistent with that notion. Heightened attention was therefore dedicated to understand the mechanism of action of the Ag/Zn redox couple.

Electron paramagnetic resonance studies provided conclusive evidence that WED does host an active redox couple that is capable of reducing molecular oxygen to superoxide anion radical. In aqueous environment, superoxides would be expected to rapidly dismutate to form hydrogen peroxide. In the presence of metal ions in the medium, the formation of hydroxyl radicals from superoxide as well as hydrogen peroxide is also expected. Blunting of the EPR spectra in the presence of catalase and SOD therefore, supports that WED is a spontaneous source of superoxide. This is a particularly important finding because superoxide generation is at the root of the so called “respiratory burst” mounted by neutrophils at the wound site to kill *P*. *aeruginosa* and may be applicable to other types of wound bacteria [67]. The ability of a topical wound dressing to spontaneously deliver low levels of superoxide to the wound should help maintain a pathogen free wound environment. Taken together with our previously reported observation that WED improves wound closure [35], it is reasonable to conclude that the superoxide generation property of WED is not toxic for the host tissue.

Superoxide anion radical is a potent reductant [68,69]. WED, a superoxide source, may reduce and thus inactivate the Mex efflux pump system. MexR and MexT are two multidrug efflux regulators in *P*. *aeruginosa* which on oxidation, forms intermolecular disulfide bonds to dissociate from promoter DNA and derepress mexAB-oprM and mexEF-oprN respectively, while in a reduced state they do not transcribe the operons. [51,52]. In this way, WED may help overcome bacterial antibiotic resistance that heavily relies on Mex efflux pump activity [70,71]. The pathogenicity of bacteria relies on quorum sensing (QS) [72]. In *P*. *aeruginosa*, QS is primarily regulated by *las* and *rhl* [73,74]. *P*. *aeruginosa* that are defective in QS are compromised in their ability to form biofilms [75]. QS inhibitors increase the susceptibility of biofilm to multiple types of antibiotics [75]. LasR and RhlR also drive the biosynthesis of pyocyanin by binding directly to the phenazine biosynthetic operons [58]. Pyocyanin is recognized as an electron shuttle for bacterial respiration and has a significant role in *P*. *aeruginosa* biofilm formation by acting as the terminal signaling factor in the quorum sensing network [76,77]. The complete virulence of *P*. *aeruginosa* can only be experienced when pyocyanin is produced [78].

Additionally, our observation that WED inhibits glycerol-3-phosphate dehydrogenase (G3P-dehydrogenase) activity is consistent with the notion that electric fields may influence molecular charge distributions on enzymes, particularly membrane enzymes, and interfere with enzymatic activity [79–81]. G3P-dehydrogenase is involved in respiration, glycolysis, and phospholipid biosynthesis and also participates in persister cell formation, which are dormant, differentiated spore-like form of bacteria that can survive after extreme antibiotic treatment [59,60].

Taken together, Ag/Zn WED represents a novel therapeutic platform that may improve wound closure by improving re-epithelialization and by disrupting *P*. *aeruginosa* bacterial biofilm infection. Safety in patients, low cost and long shelf-life are some of the attractive practical advantages of WED that warrant further *in vivo* studies.
